# Reducing health inequity in Indonesia through a comprehensive training on social determinants of health among researchers and policy makers

**DOI:** 10.1186/1471-2458-14-S1-O2

**Published:** 2014-01-29

**Authors:** Dwidjo Susilo, Malin Eriksson, Raman Preet, Siwi Padmawati, Istiti Kandarina, Laksono Trisnantoro, John Kinsman

**Affiliations:** 1 Universitas Muhammadiyah Jakarta, Jakarta 15419, Indonesia; 2 Umea University, 90187 Umea, Sweden; 3 Universitas Gadjah Mada, Yogyakarta 55281, Indonesia

## Background

Indonesia is a country with great disparities, geographically, demographically, and economically. The implementation of decentralisation had a tremendous impact on the national health system. Since the effective development and implementation of all policies depended on the capability of the heads of districts, these conditions influenced the health of the Indonesian people. In addition, it also brought about health inequity in Indonesia. This study aimed to provide a situation analysis for Indonesia, focusing on the current social determinants of health-related (SDH-related) training in Indonesia, and gaps identified.

## Materials and methods

Information on SDH-related curricula at public health schools was collected through using search engines on the internet. We also interviewed 15 key informants at national and local levels to develop a better insight of SDH issues in Indonesia. They were categorised as decision-makers, donors, NGOs, WHO, and SDH experts. Informants were interviewed using a ‘category-specific’ interview guide that was produced by a team at Umea University.

## Results

The terms ‘social determinants of health’ (SDH) were not widely used or understood in Indonesia. SDH was not taught explicitly in any graduate schools of public health in Indonesia. SDH was taught as only a component of different courses. There were also a very limited number of seminars on SDH in Indonesia. The knowledge of SDH was very inadequate, but not limited among those who work in the health sector, but also those working in other sectors. National level data and regulations were insufficient to effectively address SDH, but rather data and interventions are needed at district level.

## Conclusions

There are currently no SDH-specific courses available, although SDH-related topics are included in some of the existing courses at the public health schools. Intensive and more structured training on SDH is needed in order to ensure a good understanding of SDH in Indonesia among key research and policy stakeholders in all sectors and at all levels.

